# Supplementary material for: The shifting landscape of private healthcare providers before and during the COVID-19 pandemic: Lessons to strengthen the private sectors engagement for future pandemic and tuberculosis care
Source: PLOS Glob Public Health. 2024 Oct 3;4(10):e0003112. doi: 10.1371/journal.pgph.0003112 (PMC11449363; doi:10.1371/journal.pgph.0003112)
Supplement: S1 Fig — (DOCX) [file pgph.0003112.s002.docx]

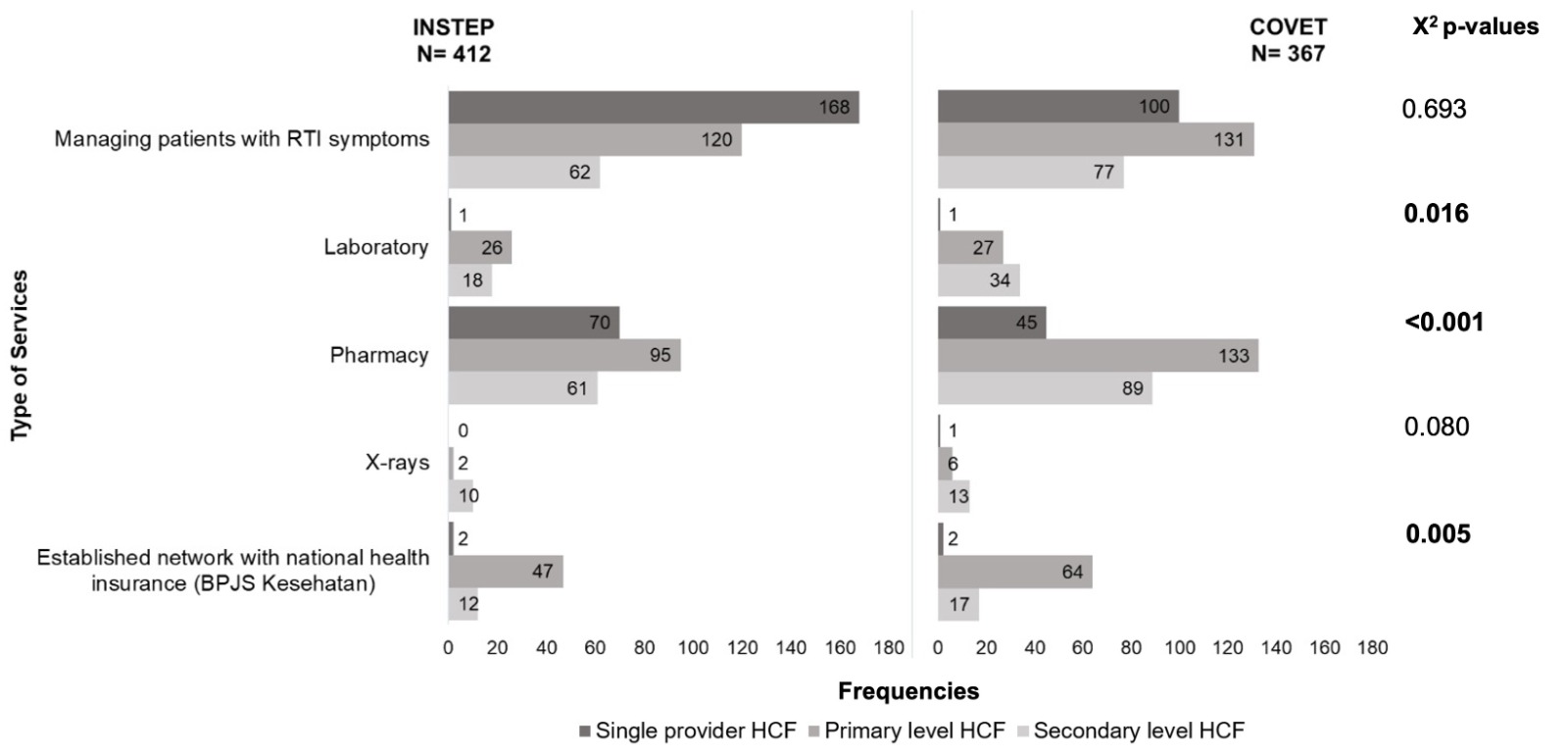


**S1 Fig. Changes in services provided by private healthcare facilities interviewed during INSTEP and COVET studies stratified by types of healthcare facilities.** X^2^ tests were performed to compare the overall proportion of HCFs (i.e., not stratified according to HCF types) with specified services; p-values <0.05 indicate significant relative change (in %).
