# Supplementary material for: The shifting landscape of private healthcare providers before and during the COVID-19 pandemic: Lessons to strengthen the private sectors engagement for future pandemic and tuberculosis care
Source: PLOS Glob Public Health. 2024 Oct 3;4(10):e0003112. doi: 10.1371/journal.pgph.0003112 (PMC11449363; doi:10.1371/journal.pgph.0003112)
Supplement: S2 Fig — (DOCX) [file pgph.0003112.s003.docx]

**
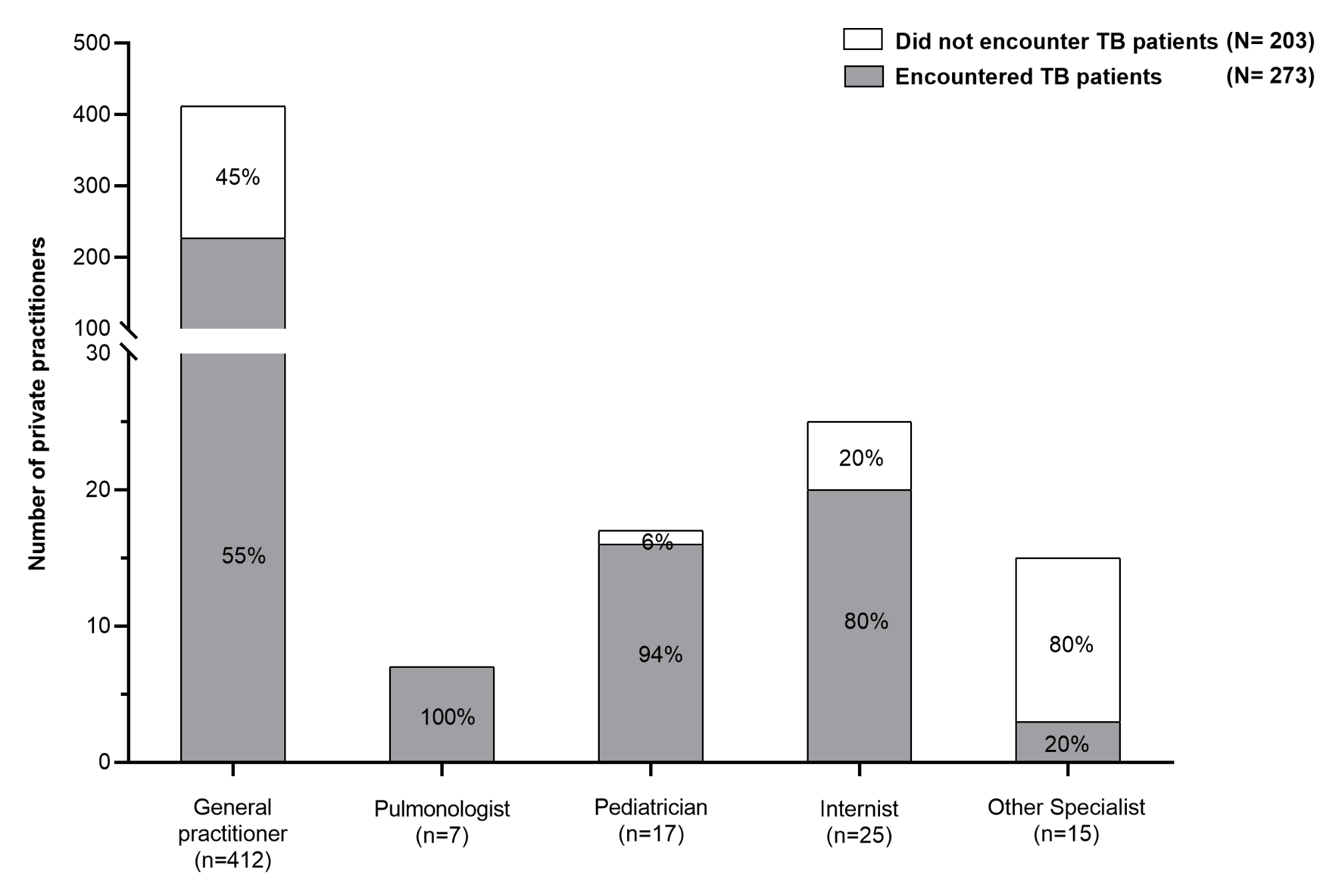
**

**S2 Fig. Qualifications and proportion of private practitioners in COVET study who managed patients with respiratory tract infection (RTI) symptoms included and those who encountered TB patients (N=476)**
