# Supplementary material for: The shifting landscape of private healthcare providers before and during the COVID-19 pandemic: Lessons to strengthen the private sectors engagement for future pandemic and tuberculosis care
Source: PLOS Glob Public Health. 2024 Oct 3;4(10):e0003112. doi: 10.1371/journal.pgph.0003112 (PMC11449363; doi:10.1371/journal.pgph.0003112)
Supplement: S1 Table — (DOCX) [file pgph.0003112.s004.docx]

**S1 Table. Characteristics of private practitioners who managed patients with RTI symptoms in INSTEP (2017) and COVET (2021) studies**

| **Characteristics** | **INSTEP Study**  **(n=594)**  **n (%)** | **COVET Study**  **(n=476)**  **n (%)** | ***p*-value**^†^ |
| --- | --- | --- | --- |
| Age, years, *median (IQR)*  *Missing* | 39 (29 - 52) | 38 (29 - 50) | **<0.001** |
| Male | 113 | 13 |  |
| Managed patients with RTI symptoms | 225 (37.9) | 183 (38.4) | 0.849 |
| Encountered TB patients |  |  |  |
| **Qualification** |  |  |  |
| General Practitioner | 474 (79.8) | 412 (86.6) | **0.003** |
| Specialist | 120 (20.2) | 64 (13.4) | N/A^‡^ |
| *Pulmonologist* | 0/120 (0.0) | 7/64 (11.0) |  |
| *Pediatrician* | 25/120 (20.8) | 17/64 (26.6) |  |
| *Internist* | 21/120 (17.5) | 25/64 (39.0) |  |
| **Number of doctors practicing in HCFs** |  |  |  |
| Single provider HCF^*^ | 130 (21.9) | 79 (16.6) | **0.029** |
| Multiple provider HCF | 464 (78.1) | 397 (83.4) |  |
| *Primary level HCF*^**^ | 291/464 (62.7) | 275/397 (69.3) | **0.043** |
| *Secondary level HCF*^***^ | 173/464 (37.3) | 122/397 (30.7) |  |
| **Schedule of Practice** |  |  |  |
| Weekdays only | 377 (63.5) | 238 (50.0) | **<0.001** |
| Weekend only | 23 (3.9) | 20 (4.2) |  |
| Both weekdays and weekend | 174 (29.3) | 184 (38.7) |  |
| By appointment only | 18 (3.0) | 34 (7.1) |  |
| *Missing* | 2 | 0 |  |
| Time of practice, total days per week, *median (IQR)* | 4 (2 – 5) | 3 (2 - 5) |  |

^*^Healthcare facilities (HCFs) with healthcare/services provided by a single general practitioner

^**^Healthcare facilities (HCFs) with healthcare/services provided by at least two general practitioners

^***^Healthcare facilities (HCF) with healthcare/services provided by at least one specialist and other specialist(s)/general practitioner(s)

^†^p-values were calculated using chi-square tests unless indicated otherwise

^‡^We did not show the p-value here as we were not trying to make a statistical inference since no pulmonologist were successfully interviewed during INSTEP study

Abbreviations:

COVET – COVID Impact on Private Health Markets; INSTEP – Investigation of Health Services for TB by External Private Providers; HCF – Healthcare facility; IQR – Interquartile Range (25 – 75); RTI – Respiratory Tract Infection; TB – Tuberculosis

**Bold** indicates that the finding is statistically significant with α≤0.05
