# Supplementary material for: The shifting landscape of private healthcare providers before and during the COVID-19 pandemic: Lessons to strengthen the private sectors engagement for future pandemic and tuberculosis care
Source: PLOS Glob Public Health. 2024 Oct 3;4(10):e0003112. doi: 10.1371/journal.pgph.0003112 (PMC11449363; doi:10.1371/journal.pgph.0003112)
Supplement: S2 Table — (DOCX) [file pgph.0003112.s005.docx]

**S2 Table (Extension of Table 2). Impact of COVID-19 pandemic on services offered at private healthcare facilities in Bandung stratified by types of healthcare providers during COVET (N= 235)^§^**

| **Characteristics** | **Single provider HCF***  **(n=78)**  **n (%)** | **Primary level HCF****  **(n=109)**  **n (%)** | **Secondary level HCF*****  **(n=48)**  **n (%)** |
| --- | --- | --- | --- |
| **Changes in number of visiting patients per day** |  |  |  |
| No | 10 (12.8) | 8 (7.3) | 11 (22.9) |
| Yes | 64 (82.1) | 94 (86.3) | 34 (70.8) |
| *More patients* | 14/64 (21.9) | 32/94 (34.0) | 4/34 (11.8) |
| *Fewer patients* | 50/64 (78.1) | 62/94 (66.0) | 30/34 (88.2) |
| Not sure | 4 (5.1) | 7 (6.4) | 3 (6.3) |
| **Changes in number of services offered** |  |  |  |
| No | 63 (80.8) | 73 (67.0) | 36 (75.0) |
| Yes | 15 (19.2) | 36 (33.0) | 12 (25.0) |
| *More services* | 6/15 (40.0) | 14/36 (38.9) | 6/12 (50.0) |
| *Fewer services* | 6/15 (40.0) | 16/36 (44.4) | 3/12 (25.0) |
| *Both*^†^ | 3/15 (20.0) | 6/36 (16.7) | 3/12 (25.0) |
| **Services that were no longer offered due to COVID-19 pandemic** |  |  |  |
| Nebulizer | 1 (1.3) | 10 (9.2) | 4 (8.3) |
| Dental care | 0 (0.0) | 3 (2.8) | 0 (0.0) |
| Home visit | 1 (1.3) | 3 (2.8) | 1 (2.1) |
| Spirometry | 0 | 1 (0.9) | 1 (2.1) |
| **Change in registration fees** |  |  |  |
| No | 77 (98.7) | 99 (90.8) | 42 (87.5) |
| Yes | 1 (1.3) | 1 (0.9) | 0 (0.0) |
| Not sure | 0 (0.0) | 9 (8.3) | 6 (12.5) |
| **Change in consultation fees** |  |  |  |
| No | 75 (96.2) | 94 (86.2) | 42 (87.5) |
| Yes | 2 (2.6) | 5 (4.6) | 1 (2.1) |
| Not sure | 1 (1.3) | 10 (9.2) | 5 (10.4) |
| **Changes in patient demographic during COVID-19 pandemic** |  |  |  |
| No | 60 (76.9) | 85 (78.0) | 36 (75.0) |
| Yes | 11 (14.1) | 6 (5.5) | 3 (6.2) |
| *Patients were older* | 3/11 (27.3) | 2/6 (33.3) | 0/3 (0.0) |
| *Patients were younger* | 2/11 (18.2) | 1/6 (16.7) | 2/3 (66.7) |
| *Patients were sicker than before* | 5/11 (45.5) | 4/6 (66.7) | 1/3 (33.3) |
| *Patients were unemployed* | 4/11 (36.4) | 0/6 (0.0) | 1/3 (33.3) |
| *Patients were do not have insurance* | 5/11 (45.5) | 0/6 (0.0) | 0/3 (0.0) |
| *Patients were more from urban areas than before* | 1/11 (9.1) | 1/6 (16.7) | 2/3 (66.7) |
| *Patients were more from rural areas than before* | 1/11 (9.1) | 0/6 (0.0) | 0/3 (0.0) |
| *Others*^‡^ | 0/11 (0.0) | 0/6 (0.0) | 1/3 (33.3) |
| Don't know | 7 (9.0) | 18 (16.5) | 9 (18.8) |

^*^Healthcare facility (HCF) with healthcare/services provided by a single general practitioner

^**^Healthcare facility (HCF) with healthcare/services provided by at least two general practitioners

^***^Healthcare facility (HCF) with healthcare/services provided by at least one specialist and other specialist(s)/general practitioner(s)

^†^Several services were being added but several others were no longer offered

^‡^Other changes in patients’ demographic during COVID-19 pandemic

**^§^**Data were obtained from interviews with doctors managing patients with RTI symptoms, representing each healthcare facilities included in COVET study

Abbreviation:

HCF – Healthcare facility; PPs – Private Practitioners
