# Supplementary material for: The shifting landscape of private healthcare providers before and during the COVID-19 pandemic: Lessons to strengthen the private sectors engagement for future pandemic and tuberculosis care
Source: PLOS Glob Public Health. 2024 Oct 3;4(10):e0003112. doi: 10.1371/journal.pgph.0003112 (PMC11449363; doi:10.1371/journal.pgph.0003112)
Supplement: S3 Table — (DOCX) [file pgph.0003112.s006.docx]

**S3 Table. Characteristics of practicing private practitioners in INSTEP and COVET studies**

| \| **Characteristics** \| **INSTEP Study**  **(n=936)**  **n (%)** \| **COVET Study**  **(n=872)**  **n (%)** \| ***p*-value**^†^ \| \| --- \| --- \| --- \| --- \| \| Age, years, *median (IQR)*  *Missing* \| 40 (30 - 53)  *399* \| 40 (31 - 51)  *229* \| 0.824 000 \| \| Male \| 305 (32.6) \| 369 (42.3) \| **<0.001** \| \| **Qualification** \|  \|  \|  \| \| General Practitioner \| 651 (69.6) \| 546 (62.6) \| **<0.001** \| \| Specialist \| 285 (30.4) \| 270 (31.0) \|  \| \| *Pulmonologist* \| 2/285 (0.7) \| 8/270 (3.0) \| 0.075^‡^ \| \| *Pediatrician* \| 44/285 (15.4) \| 33/270 (12.2) \|  \| \| *Internist* \| 38/285 (13.3) \| 40/270 (14.8) \|  \| \| *Missing* \| 0 \| 56 \|  \| \| **Number of doctors practicing in HCFs** \|  \|  \|  \| \| Single provider HCF^*^ \| 186 (19.9) \| 132 (15.1) \| **0.008** \| \| Multiple provider HCF \| 750 (80.1) \| 740 (84.9) \|  \| \| *Primary level HCF*^**^ \| *399 (53.2)* \| *381 (51.5)* \| 0.507 \| \| *Secondary level HCF*^***^ \| *351 (46.8)* \| *359 (48.5)* \|  \|   ^*^Healthcare facility (HCF) with health care/services provided by a single general practitioner  ^**^Healthcare facility (HCF) that providing essential services like acute illness, injuries, or screenings and provided by at least two general practitioners  ^***^Healthcare facility (HCF) with health services provided by at least one specialist and other specialist(s)/general practitioner(s)  ^†^p-values were calculated using chi-square tests unless indicated otherwise  ^‡^ p-value 0.075 only display for Pulmonologist, Pediatrician, and Internist  Abbreviations:  COVET – COVID Impact on Private Health Markets; HCF – Healthcare facility; INSTEP – Investigation of Health Services for TB by External Private Providers; IQR – Interquartile Range (25 – 75)  **Bold** indicates that the finding is statistically significant with α≤0.05 |  |  |
| --- | --- | --- | --- | --- | --- | --- | --- | --- | --- | --- | --- | --- | --- | --- | --- | --- | --- | --- | --- | --- | --- | --- | --- | --- | --- | --- | --- | --- | --- | --- | --- | --- | --- | --- | --- | --- | --- | --- | --- | --- | --- | --- | --- | --- | --- | --- | --- | --- | --- | --- | --- | --- | --- | --- | --- | --- | --- | --- | --- | --- | --- | --- |
