# Supplementary material for: The shifting landscape of private healthcare providers before and during the COVID-19 pandemic: Lessons to strengthen the private sectors engagement for future pandemic and tuberculosis care
Source: PLOS Glob Public Health. 2024 Oct 3;4(10):e0003112. doi: 10.1371/journal.pgph.0003112 (PMC11449363; doi:10.1371/journal.pgph.0003112)
Supplement: Attachment — Submitted filename: Response to correction 30Jul2024.docx [file pgph.0003112.s007.docx]

Letter of Response

To PLOS Global Public Health

Submission PGPH-D-24-00569R1

- [EMID:7addbd5c4821de22]

Bandung 1^st^ August 2024

Dear Editor

Thank you for your correction. We have corrected our manuscript entitled "The shifting landscape of private healthcare providers before and during the COVID-19 pandemic: lessons to strengthen the private sectors engagement for future pandemic and tuberculosis care" on the points you’ve raised.

In detailed:

1. Please include a complete copy of PLOS’ questionnaire on inclusivity in global research in your revised manuscript.

We have filled in the questionnaire, and it is already downloaded at the PLOS Site.

2. We noticed that you used “data not shown” in the manuscript. We do not allow these references, as the PLOS data access policy requires that all data be either published with the manuscript or made available in a publicly accessible database. Please amend the supplementary material to include the referenced data or remove the references.

Thank you for your correction. We have amended the text in line number 695, delete the “data not shown” and change to “….(Table 1 and S3 Table)”. Additionally, we have added supplement table S3 to support the information.

3. Some material included in your submission may be copyrighted. According to PLOS’s copyright policy, authors who use figures or other material (e.g., graphics, clipart, maps) from another author or copyright holder must demonstrate or obtain permission to publish this material under the Creative Commons Attribution 4.0 International (CC BY 4.0) License used by PLOS journals.

The basic map that we use for this figure acquired from an open source with a link http://satudata.bandung.go.id/dataset/batas-administrasi-kelurahan-kota-bandung. We have already added in the reference number 26 for this purpose and added the source link at the map.

Thank you

Best regads

Bachti Alisjahbana
